# Diagnostic value of the cerebrospinal fluid lipoarabinomannan assay for tuberculous meningitis: a systematic review and meta-analysis

**DOI:** 10.3389/fpubh.2023.1228134

**Published:** 2023-09-21

**Authors:** Ya-Li Chen, Meng-Meng Zhu, Cui-Ping Guan, Yan-An Zhang, Mao-Shui Wang

**Affiliations:** ^1^Department of Lab Medicine, Shandong Public Health Clinical Center, Shandong University, Jinan, China; ^2^Shandong Key Laboratory of Infectious Respiratory Disease, Jinan, China; ^3^Department of Cardiovascular Surgery, Shandong Public Health Clinical Center, Shandong University, Jinan, China

**Keywords:** tuberculous meningitis, lipoarabinomannan, diagnosis, meta-analysis, cerebrospinal fluid

## Abstract

**Objective:**

This systematic review aims to evaluate the diagnostic accuracy of cerebrospinal fluid (CSF) lipoarabinomannan (LAM) assays in detecting tuberculous meningitis (TBM).

**Methods:**

A systematic review search was conducted in PubMed and five other databases up to April 2023. Studies that evaluated the diagnostic accuracy of CSF LAM assays were included with either definitive or composite reference standard used as the preferred reference standard. The quality of the included studies was assessed using the QUADAS-2 tool. We performed a bivariate random-effects meta-analysis and calculated the summary diagnostic statistics.

**Results:**

A total of six studies, including a sample size of 999, were included in the final analysis. The pooled sensitivity, specificity, and area under the receiver operating characteristic curve (AUC) of CSF LAM for diagnosing TBM were determined to be 0.44 (95% CI: 0.31–0.58), 0.89 (95% CI: 0.81–0.93), and 0.76 (95% CI: 0.73–0.80), respectively. Significant heterogeneity was observed in both sensitivity (*Q* = 73.82, *p* < 0.01; *I*^2^ = 86.45, 95%CI: 79.64–93.27) and specificity (*Q* = 95.34, *p* < 0.01; *I*^2^ = 89.51, 95% CI: 84.61–94.42). Regression analysis indicated that the study design (retrospective vs. prospective) was associated with the heterogeneity of pooled sensitivity and specificity (all *p* < 0.05).

**Conclusion:**

Although more prospective studies are required to validate the role of the CSF LAM assay, current evidence supports that the performance of the CSF LAM assay is unsatisfactory for the TBM diagnosis. Additionally, the optimization of the CSF LAM assay (e.g., improvements in CSF collection and preparation methods) should be considered to improve its performance.

## Introduction

Tuberculous meningitis (TBM) is a serious disease with a fatality rate as high as half of all cases (1). A delay in the diagnosis of TBM is a key factor contributing to poor outcomes. Most microbiological examinations have low sensitivity, making them inadequate for TBM diagnosis; however, other examinations are unspecific and do not yield satisfactory results (2, 3). Furthermore, diagnostic delays are largely associated with a lack of practical TBM diagnostic criteria. During the last decade, a novel diagnostic scoring system for TBM was developed by Marais et al. (4). However, a significant number of confirmed TBM cases had low scores and were classified as possible TBM (3). Therefore, there remains a need to develop new diagnostic tools for TBM.

Lipoarabinomannan (LAM) is a structurally important 17.5 kD heat-stable glycolipid found in the cell walls of *Mycobacterium tuberculosis*. It can account for up to 15% of the total bacterial weight and serves as an immunogenic virulence factor released by metabolically active or degrading bacterial cells during TB infection (5, 6). Currently, commercial urinary LAM assays are widely used. The urinary LAM assay has several advantages, making it attractive for diagnosing TB. However, several disadvantages have been observed according to the available systematic reviews. For example, it has suboptimal sensitivity for routine clinical use, ranging from 13% to 93% (7). Furthermore, the difference in sensitivity between different commercial assays is significant (8–10). Additionally, immunocompromised status (such as HIV status and CD4 count) is associated with the diagnostic yield of the LAM assay (8, 11).

Thus, recent studies on LAM detection have highlighted its potential diagnostic usefulness for TBM. Testing for LAM in cerebrospinal fluid (CSF) samples may provide significant clinical benefits. However, to date, no systematic review of the current evidence on CSF LAM assays for TBM diagnosis has been conducted. Hence, we reviewed diagnostic studies and analyzed the accuracy of the LAM antigen in CSF samples for the diagnosis of TBM.

## Methods

### Search strategy

The literature search was performed on 19 April 2023. Databases, specifically PubMed, Embase, Scopus, Web of Science, CINAHL, and the Cochrane Library, were searched using a defined search strategy outlined in the online Supplementary material. The search terms included “CSF,” “TBM,” and “diagnosis.” The systematic review was performed following the Preferred Reporting Items for Systematic Reviews and Meta-Analyses guidelines (12), and the protocol was registered in PROSPERO (CRD42023417827).

### Eligibility

Original studies that reported the diagnostic accuracy of CSF LAM assays for TBM were included in the study. Only English literature was included, but time restrictions were not imposed. We excluded duplicates, animal studies, bench studies, case reports (less than 10 cases), chapters, conference abstracts, editorials, comments, and reviews. The eligibility of the study was assessed by two investigators (CYL and ZMM) who independently screened titles and abstracts, which was followed by a full-text review. A third reviewer (GCP) determined eligibility in the event of any discrepancies.

The diagnostic criteria for TBM include both definite and clinical diagnoses. The definite TBM diagnosis is made based on histological examination, acid-fast bacilli (AFB) smear, TB-PCR, or culture using central nervous system (CNS) samples. A clinical diagnosis (probable or possible TBM) is made following a composite reference standard (CRS), which outlines symptoms, abnormal CSF features, abnormal CNS radiological evidence, or non-CNS TB evidence. The control subjects were healthy subjects or non-TBM patients. The cutoff value for urinary LAM assays was used to determine the positive/negative events for CSF LAM assays.

### Bias assessment

The included studies were critically appraised by two independent reviewers (CYL and ZMM) for methodological quality in the review using the Quality Assessment of Diagnostic Accuracy Studies (QUADAS-2) tool (13). This tool allows for the review of individual studies for potential sources of bias and concerns regarding applicability. QUADAS-2 consists of four domains: patient selection, index test, reference standard, and flow and timing. All four domains for potential risk of bias and the first three domains for concerns with applicability must be assessed. The review was guided by structured questions, and all domains of each included study were rated as high, low, or unclear risk. RevMan (version 5.3) was used for visualization.

### Data collection

Data collection was performed independently by two reviewers (Y-LC and M-MZ), and the following variables were collected: first author, publication year, country, sample size, TBM diagnostic criteria, controls, sex, age, HIV status, CD4, LAM assays (principle and cutoff values), and diagnostic performance (true positive, false positive, true negative, and false negative events). Any discrepancy that arose between the reviewers was resolved by the third reviewer (C-PG).

### Statistical analysis

Statistical analysis was performed using Stata software (Version 15.0; Stata Corporation, College Station, TX). The pooled sensitivity, specificity, area under the curve (AUC), and corresponding 95% confidence intervals (CIs) were assessed. Heterogeneity was statistically assessed using the Q test and *I*^2^. A random effect model was used for estimation. Meta-regression analyses were implemented independently for each variable, and the following variables were taken into consideration: study design, definite or CRS TBM, HIV status, CSF collection, unprepared or supernatant CSF, enzyme-linked immunosorbent assay (ELISA), or lateral flow (LF) assay. Publication bias was evaluated using Deeks’ funnel plot asymmetry test. A *p*-value of less than 0.05 was considered statistically significant.

## Results

### Literature selection

**Figure 1:**
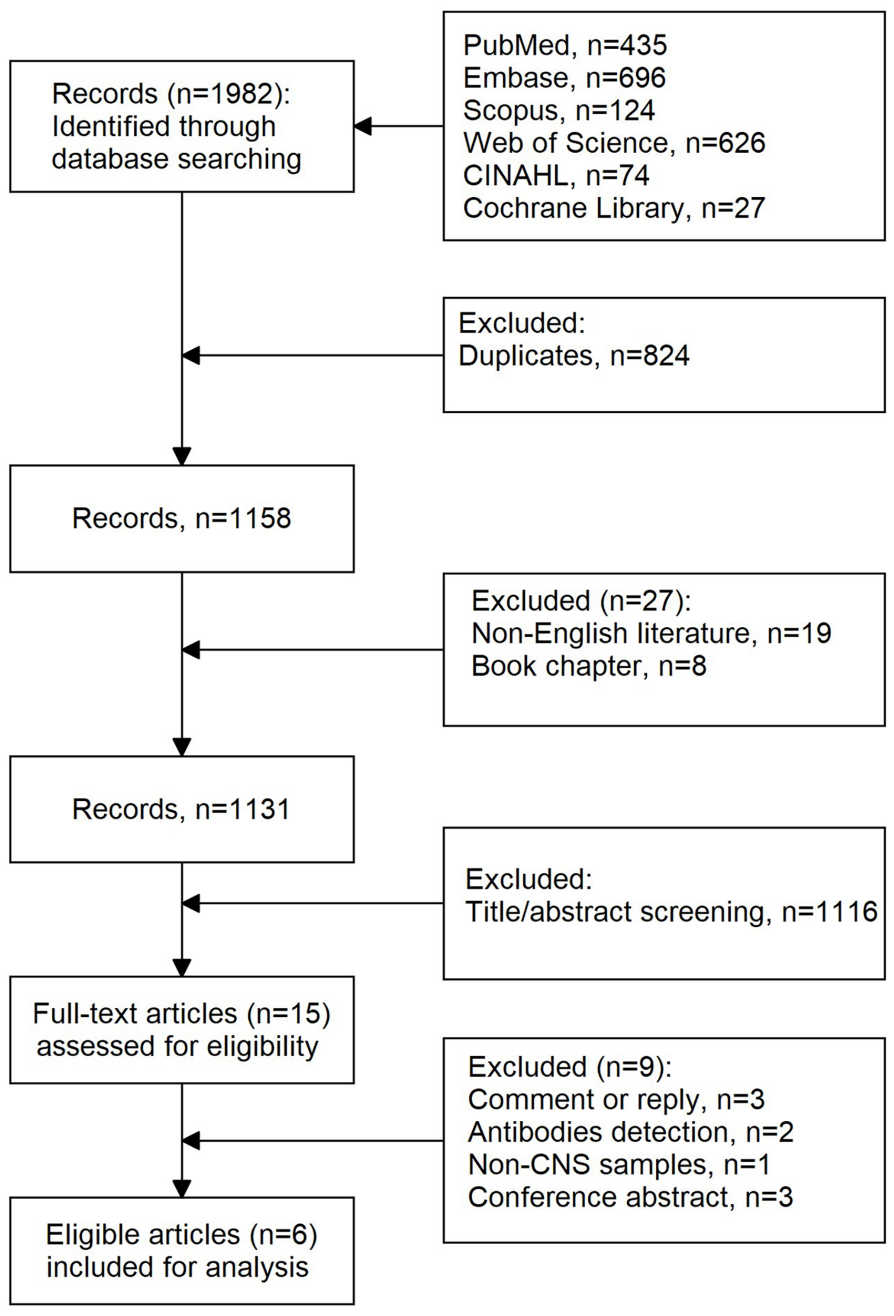
Flow chart of literature selection.

A total of 1,982 records were retrieved from the five databases. Figure 1 presents the literature selection process. First, duplicates (*n* = 824) were identified using EndNote software and removed from the list. Second, non-English literature (*n* = 19) and studies without original data (*n* = 8) were excluded. Two independent reviewers screened the titles and abstracts, and 1,116 records were removed. Finally, 15 full-text studies were assessed for eligibility. Nine studies were excluded because they were comments, replies (*n* = 3), or conference abstracts (*n* = 3) and because they featured antibody detection (*n* = 2) and non-CNS samples (*n* = 1). Therefore, six studies (999 cases) were included in the final analysis.

### Study characteristics

Table 1 presents the baseline characteristics of the studies. All studies (*n* = 6) were published after 2009 and conducted in African countries, including South Africa (*n* = 2) (14, 15), Uganda (*n* = 3) (16, 17, 19), and Zambia (*n* = 1) (18). Three (14, 16, 17) were designed retrospectively and others (*n* = 3) prospectively. Only one study had a study period of less than 1 year (16). Definite TBM was defined in all six studies, and clinical diagnoses were defined in five studies (14–17, 19). In five studies, CSF samples were collected by lumbar puncture, and in the remaining study (*n* = 1) (16), they were collected from the fourth ventricle. Unprepared CSF samples were evaluated in all six studies, and only one study evaluated the CSF supernatant (16). The ELISA method for LAM measurement was evaluated in three studies (14–16), and the lateral flow assay was evaluated in four studies (16–19). The cutoff values of urinary LAM assays were assessed in all studies. In addition, receiver operating characteristic (ROC) curve analysis was used to determine the optimal cutoff value in two studies (14, 15).

**Table 1:** The characteristics of the included studies in the meta-analysis.

Study characteristics					Patient characteristics							CSF LAM assays					Diagnostic performance			
Sequence	First author, year	Country	Study period	Study design	Subjects (*n*)	Age (median, IQR)	Female (%)	HIV status (+; %, *n*/*N*)	CD4 cell count (median, IQR)	TBM diagnostic criteria (*n*)	Non-TBM (*n*)	CSF collection	CSF samples	LAM assay	Manufacturer	Cut-off value	TP (*n*)	FP (*n*)	FN (*n*)	TN (*n*)
1.1	Vinod B Patel, 2009 (14)	South Africa	2004.01–2005.12	Retrospective	50	30.3	60%	68% (34/50)	–	37 (definite, *n* = 14; probable, *n* = 23)	13 (cryptococcal meningitis, *n* = 4; cerebral toxoplasmosis, *n* = 2; viral meningitis, *n* = 5; acute demyelinating encephalomyelitis, *n* = 1; and epilepsy, *n* = 1)	Lumbar puncture	Unprepared CSF	ELISA	Clearview TB (Inverness)	0.1 (increase, standard)	15	5	22	8
1.2										14 (definite)							9	–	5	–
1.3										23 (probable)							6	–	17	–
1.4										37 (definite, *n* = 14; probable, *n* = 23)						0.22 (increase, ROC analysis)	14	4	23	9
1.5										14 (definite)							9	–	5	–
1.6										23 (probable)							5	–	18	–
2.1	Vinod B. Patel, 2010 (15)	South Africa	2008.01–2009.04	Prospective	148	33.5	–	84% (126/150)	84 (53–173)	94 (definite, *n* = 39; probable, *n* = 55)	54 (cryptococcal meningitis, *n* = 30; bacterial meningitis, *n* = 5; viral meningitis, *n* = 14, neoplastic meningitis, *n* = 2, mucormycosis, *n* = 1; venous sinus thrombosis with CSF change, *n* = 1; and neurosyphilis, *n* = 1)	Lumbar puncture	Unprepared CSF	ELISA	Clearview TB (Inverness)	0.18 (ROC analysis)	13	3	81	51
2.2										39 (definite, *n* = 39)							12	3	27	51
2.3																0.1295 (standard)	27	19	12	35
2.4																0.148	18	6	21	48
2.5																0.18 (ROC analysis)	12	3	27	51
2.6								10 (HIV-)		-	10						–	–	–	10
2.7								81 (HIV+)		34 (definite, *n* = 38)	47						12	2	22	45
3.1	Janneke A. Cox, 2015 (16)	Uganda	2013.02–2013.06	Retrospective	91	35 (28–40)	0.57	100% (91/91)	47 (21–165)	14 (definite, *n* = 8; probable, *n* = 6)	69 (including cryptococcus neoformans meningitis, *n* = 11; bacterial meningitis, *n* = 2; candidal meningitis, *n* = 1)	4th ventricle	Unprepared CSF	LFA	AlereLAM	CU + 1	7	19	7	48
3.2																CU + 2	4	9	10	60
3.3													Supernatant			CU + 1	10	21	4	48
3.4																CU + 2	7	12	7	57
3.5										13 (definite, *n* = 7; probable, *n* = 6)				ELISA	Clearview TB (Alere)	0.1 (increase, standard)	5	6	8	63
3.6										8 (histopathologically definite)			Unprepared CSF	LFA	AlereLAM	CU + 1	6	21	2	48
3.7																CU + 2	3	9	5	60
3.8													Supernatant			CU + 1	7	21	1	48
3.9																CU + 2	6	12	2	57
3.10										7 (definite)				ELISA	Clearview TB (Alere)	0.1 (increase, standard)	3	6	4	63
3.11										22 (composite standard definite)	60	4th ventricle	Unprepared CSF	LFA	AlereLAM	CU + 1	15	13	7	47
3.12																CU + 2	9	4	13	56
3.13													Supernatant			CU + 1	17	13	5	47
3.14																CU + 2	13	6	9	54
3.15														ELISA	Clearview TB (Alere)	0.1 (increase, standard)	10	1	11	59
4.1	Richard Kwizera, 2019 (17)	Uganda	2018.04–2019.06	Retrospective	59	33 (28–40)	50%	100% (59/59)	-	17 (definite, *n* = 12; probable TBM, *n* = 5)	42 (non-TBM, *n* = 27; possible TBM, *n* = 15)	Lumbar puncture	Unprepared CSF	LFA	AlereLAM	CU + 1	4	2	13	40
4.2										12 (definite)	47 (non-TBM, *n* = 27; clinical diagnosis, *n* = 20)						4	2	8	45
5.1	Omar K. Siddiqi, 2019 (18)	Zambia	2014.04–2017.08	Prospective	550	-	47.5% (261/550)	86.4% (475/550)	-	105 (definite)	445	Lumbar puncture	Unprepared CSF	LFA	AlereLAM	CU + 1	23	26	82	419
5.2					8	25 (24–27)	3 (38)	HIV−, *n* = 8	165 (142–264)	8 (definite)	-						1	–	7	–
5.3					97	35 (30–41)	64 (66)	HIV+, *n* = 97	104 (45–167)	95 (definite)							21	–	74	–
6.1	Carson M. Quinn, 2021 (19)	Uganda	2018.05–2020.03	Prospective	101	33 (26–40)	21 (36)	94.1% (95/101)	79 (30–260)	58 (definite and probable)	43	Lumbar puncture	Unprepared CSF	LFA	FujiLAM	Standard	30	1	28	42
6.2										34 (definite)	-						25	–	9	–
6.3										24 (probable)							5	–	19	–
6.4										28 (definite or probable)							14	–	14	–
6.5										28 (definite or probable)					AlereLAM	CU + 1	4	–	24	–
6.6										17 (definite)					FujiLAM	Standard	11	–	6	–
6.7										17 (definite)					AlereLAM	CU + 1	4	–	13	–

LAM, lipoarabinomannan; ELISA, enzyme-linked immunosorbent assay; LFA, lateral flow assay; CSF, cerebrospinal fluid; TP, true positive; FP, false positive; TN, true negative; FN, false negative; CU, cutoff.

### Bias assessment

**Figure 2:**
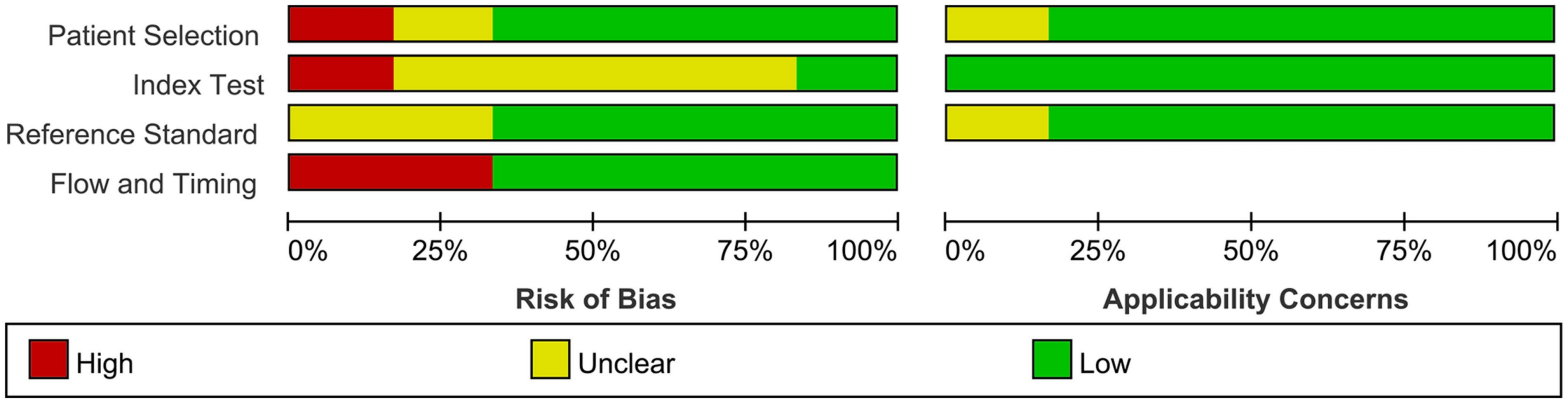
Risk of bias and applicability concerns assessed by QUADAS-2.

Figure 2 summarizes the risk of bias and the applicability of the included studies. Regarding patient selection, only one study included known microbiologically confirmed children, and the risk of bias was therefore deemed high (18). The strict diagnostic criteria indicated a high bacterial burden in the CSF samples, which would lead to an increase in the positivity of the LAM assay. A study had a high risk of bias for the index test domain because an optimal cutoff value was determined via ROC analysis, which can result in an overestimation of diagnostic performance (15). The reference method domain was at low risk of bias for all studies. For the flow and timing domains, since possible TBM was not included for analysis, the risk of bias was rated as high in one study (16), as this could result in an overestimation of the diagnostic performance of the LAM assay.

### Diagnostic performance

**Figure 3:**
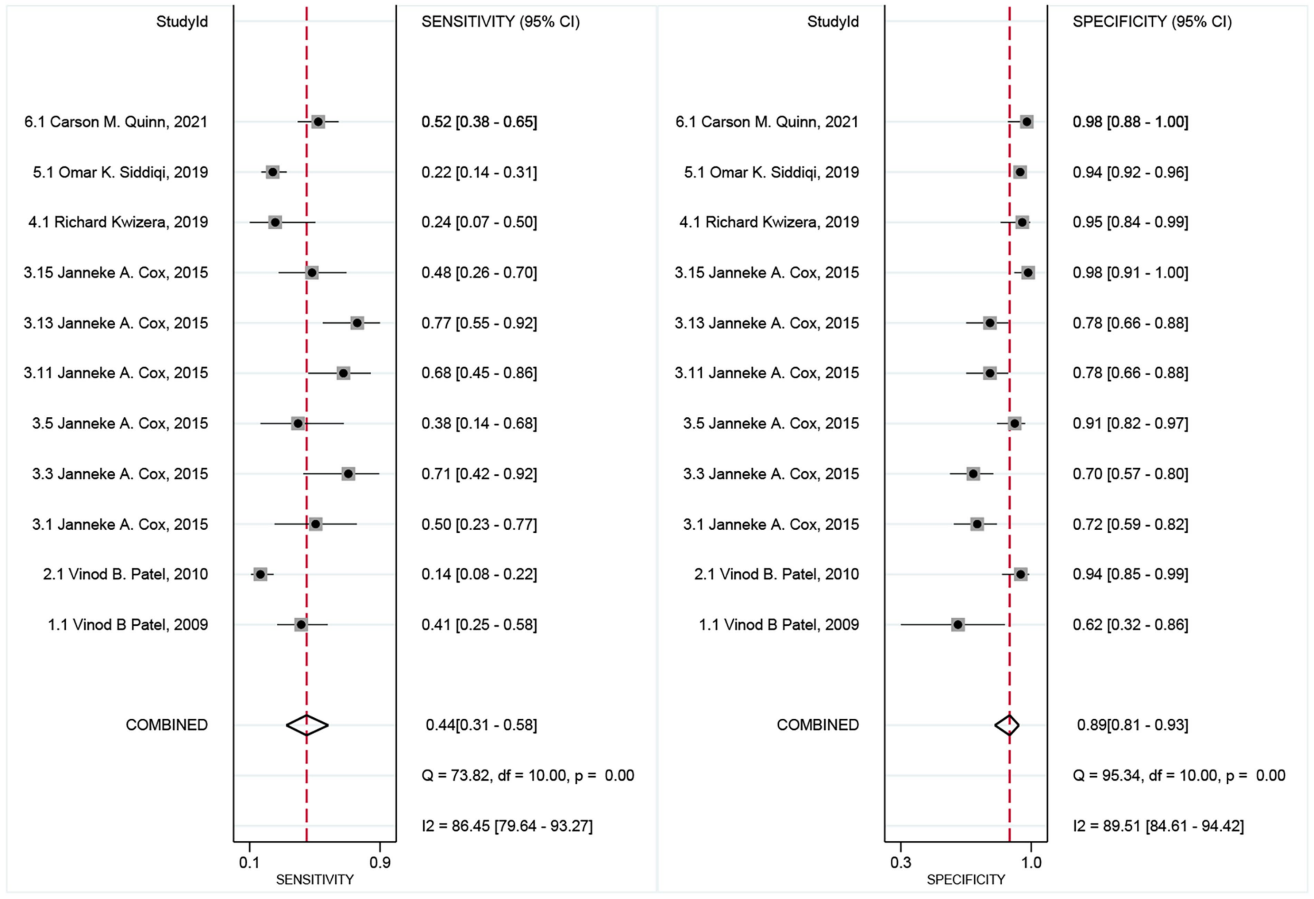
Forest plots of sensitivity and specificity for CSF LAM assay in TBM diagnosis.

**Figure 4:**
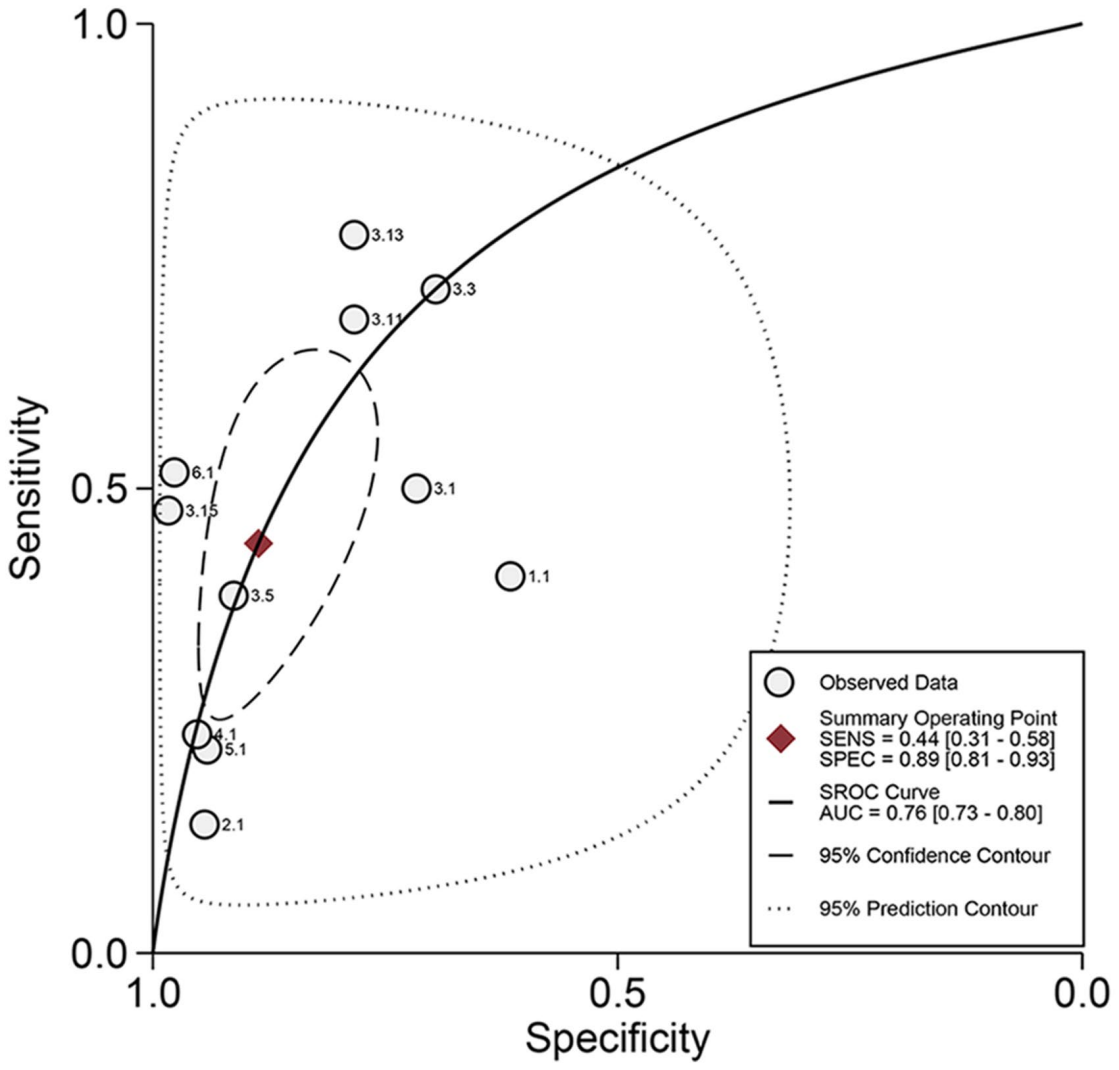
Summary receiver operating characteristic curve meta-analysis of diagnostic performance of CSF LAM assay versus composite reference standard.

**Figure 5:**
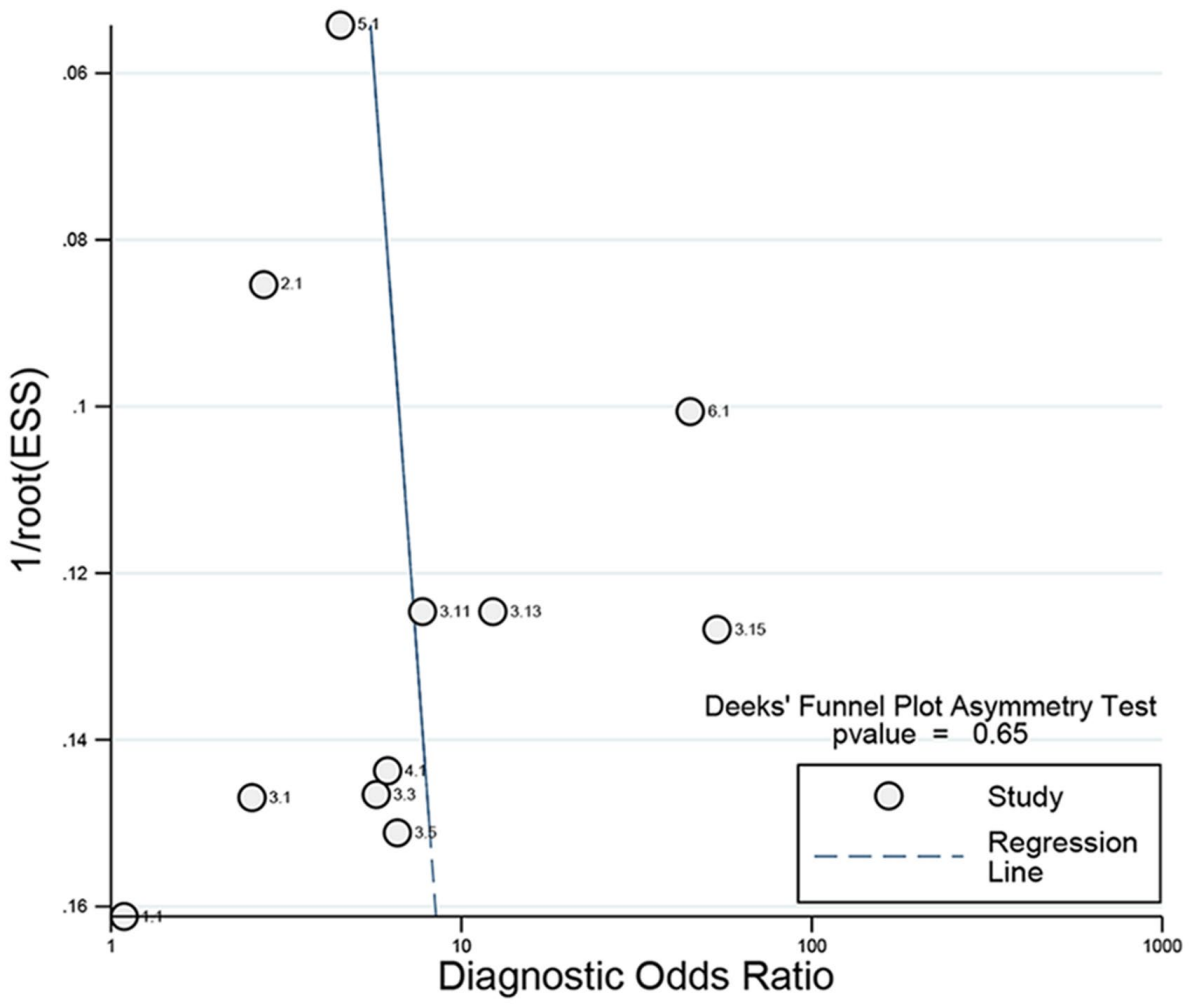
Deeks funnel plot asymmetry test to evaluate publication bias.

The pooled sensitivity (Figure 3, left), specificity (Figure 3, right), and AUC (Figure 4) of CSF LAM for TBM diagnosis were 0.44 (95% CI: 0.31–0.58), 0.89 (95% CI: 0.81–0.93), and 0.76 (95% CI: 0.73–0.80), respectively. Significant heterogeneity, which was assessed using the Q test and *I*^2^, was observed in the sensitivity (Q = 73.82, *p* < 0.01; *I*^2^ = 86.45, 95% CI: 79.64–93.27; Figure 3) and specificity (Q = 95.34, *p* < 0.01; *I*^2^ = 89.51, 95% CI: 84.61–94.42), respectively. The regression analysis revealed that the study design (retrospective vs. prospective) was associated with the heterogeneity of pooled sensitivity and specificity (all *p* < 0.05; Table 2). Deek’s plot for publication bias is presented in Figure 5. The bias test results showed no evidence of publication bias (*p* = 0.65).

**Table 2:** Meta-regression analysis of factors associated with heterogeneity of CSF LAM assays.

Variables	Category	Studies (*n*)	Pooled sensitivity		Pooled specificity		Heterogeneity (*χ*^2^)		Inconsistency (*I*^2^, 95% CI)
			Sensitivity (95%CI)	*p* value	Specificity (95%CI)	*p* value	*χ*^2^	*p* value	
Study design	Retrospective	8	0.52 (0.38–0.67)	0.04	0.84 (0.77–0.92)	0	5.34	0.07	63 (16–100)
	Prospective	3	0.27 (0.11–0.43)		0.95 (0.91–1.00)				
Diagnostic criteria	CRS (definite and clinical diagnosis)	10	0.47 (0.33–0.61)	0.17	0.88 (0.81–0.95)	0.35	1.67	0.43	0 (0–100)
	Definite	1	0.22 (−0.05–0.49)		0.94 (0.85–1.00)				
CSF collection	Lumber puncture	5	0.29 (0.17–0.40)	0.06	0.93 (0.87–0.98)	0.72	7.22	0.03	72 (39–100)
	4th ventricle	6	0.60 (0.46–0.75)		0.84 (0.74–0.94)				
CSF samples	Unprepared CSF	7	0.36 (0.22–0.51)	0.17	0.89 (0.82–0.96)	0.33	3.81	0.15	47 (0–100)
	Supernatant	4	0.61 (0.40–0.82)		0.88 (0.77–0.98)				
LAM assay	ELISA	4	0.31 (0.13–0.49)	0.36	0.92 (0.84–0.99)	0.62	2.27	0.32	12 (0–100)
	LFA	7	0.52 (0.35–0.68)		0.86 (0.78–0.95)				

LAM, lipoarabinomannan; CSF, cerebrospinal fluid; 95% CI, 95% confidence interval; ELISA, enzyme-linked immunosorbent assay; LFA, lateral flow assay; CRS, composite reference standards.

To fully outline the factors associated with heterogeneity, subgroup analyses of study design (retrospective vs. prospective), diagnostic criteria (CRS vs. definite TBM; definite vs. probable TBM), HIV status, CSF collection (lumbar puncture vs. fourth ventricle), CSF samples (unprepared vs. supernatant CSF), and LAM assay (ELISA vs. LFA) were performed subsequently, and the results are presented in Table 3. The data demonstrated that the study design, CSF collection, and CSF samples were associated with the heterogeneity of its sensitivity. Additionally, the data also supported the fact that supernatant CSF samples collected from the fourth ventricle are appropriate for CSF LAM assays.

**Table 3:** Subgroup analysis of factors associated with heterogeneity in the sensitivity of CSF LAM assays.

Variables	Category	Studies (*n*)	Sensitivity (95% CI)	*p* value
Study design	Retrospective	8	0.52 (0.39–0.66)	0.032
	Prospective	4	0.25 (0.10–0.39)	
	Overall	12	0.49 (0.38–0.60)	
Diagnostic criteria	CRS (definite and clinical diagnosis)	12	0.45 (0.31–0.59)	0.354
	Definite	8	0.55 (0.36–0.75)	
	Overall	20	0.49 (0.38–0.60)	
Diagnostic criteria	Definite	11	0.51 (0.36–0.66)	0.056
	Probable	3	0.23 (0.13–0.33)	
	Overall	14	0.44 (0.32–0.56)	
HIV status	HIV (+)	9	0.48 (0.32–0.63)	0.172
	HIV (−)	1	0.13 (−0.10–0.35)	
	Overall	10	0.44 (0.30–0.59)	
CSF collection	Lumbar puncture	6	0.27 (0.15–0.39)	0.005
	4th ventricle	6	0.60 (0.48–0.73)	
	Overall	12	0.47 (0.34–0.60)	
CSF samples	Unprepared CSF	9	0.37 (0.24–0.50)	0.047
	Supernatant CSF	5	0.65 (0.48–0.82)	
	Overall	14	0.47 (0.34–0.60)	
LAM assay	ELISA	5	0.35 (0.17–0.52)	0.287
	LFA	9	0.51 (0.33–0.69)	
	Overall	14	0.45 (0.32–0.58)	

CI, confidence interval; CRS, composite reference standard; HIV, human immunodeficiency virus; CSF, cerebrospinal fluid; LAM, lipoarabinomannan; ELISA, enzyme-linked immunosorbent assay; LFA, lateral flow assay.

## Discussion

The early diagnosis of TBM is difficult. Until recently, clear criteria for defining TBM have been lacking, and the diagnosis of TBM still largely relies on routine CSF biomarkers such as protein, white blood cell, and glucose levels. The introduction of the CSF LAM assay was revolutionary for TBM diagnosis and may address current issues in TBM diagnosis. Our study demonstrated that the CSF LAM assay had an unsatisfactory performance for TBM diagnosis, with a sensitivity, a specificity, and an AUC of 0.44 (95% CI: 0.31–0.58), 0.89 (95% CI: 0.81–0.93), and 0.76 (95% CI: 0.73–0.80), respectively. Despite a positive result, the CSF LAM assay demonstrated poor sensitivity and required further improvement.

LAM may be detected in various bodily fluids during infection as part of the mycobacterial cell wall. Therefore, it can be a promising biomarker for TB-related diseases (20). Commercial urinary LAM detection technology was developed recently, including ELISA and LFA methods (21). Previous systematic studies supported the following: (1) The pooled sensitivity of urinary LAM tests varies widely between different methods or manufacturers (22, 23); (2) Diagnostic performance is associated with HIV status and CD4+ levels (7, 8, 24); and (3) LAM assay may be considered an alternative biomarker for TB diagnosis (7) and serve as a “rule-in” test to screen for incident TB among patients with high risk.

Currently, definite cases of TBM are still difficult to diagnose, and microbiological confirmation is not always achievable. Traditional microbiological examinations, such as AFB smears, nucleic acid amplification (NAA) tests, and the mycobacterial culture of CSF samples, have been evaluated systematically. For example, an AFB smear, while quick, is highly insensitive in most settings (25). It has been reported that the CSF AFB smear has a low pooled sensitivity of 8% (95% CI: 3–21) (26). Second, the sensitivity of CSF mycobacterial culture was 29.72% (95% CI: 21.42–38.02) (1), and an important disadvantage is the slow time-to-positivity, which makes it unhelpful for the diagnosis of this rapidly progressing disease (27). Third, the pooled sensitivity and specificity of NAA tests against CRS were 68% (95% CI: 41–87) and 98% (95% CI: 95–99), respectively (28). In resource-poor settings, the availability of NAA testing is extremely limited. LAM antigen detection technology (especially the LFA method) has a short turnaround time and requires minimal training and no additional materials. Thus, CSF LAM testing may be a useful and rapid way to diagnose TBM.

In our meta-analysis, heterogeneity was assessed during the analyses, depending on whether data on variables of interest were available. Finally, five variables, such as study design, diagnostic criteria, CSF collection, CSF samples, and LAM assay, were assessed *via* meta-regression analysis, and study design is one of the factors that explain the variance in diagnostic accuracy across studies. This point was also confirmed via subgroup analysis. Moreover, the subgroup analysis also found that variation in sensitivity between studies is associated with CSF collection (lumbar puncture vs. fourth ventricle) and CSF samples (unprepared and supernatant CSF). Prior to the assay, CSF samples drained from the fourth ventricle were centrifuged at 10,000 rpm for 15 min, and the supernatant was used for CSF LAM testing. Although a previous study supported that CSF LAM positivity is associated with a positive HIV status and a low CD4 count (7), the association was not confirmed in the study, which was explained by the small sample size (HIV status) and the unavailability of data (CD4 count). The CSF LAM assay appears to have a higher sensitivity among TBM patients with HIV(+); this may be associated with the indication for the urinary LAM assay (severe HIV disease). Remarkably, a high CSF bacterial burden means an increased level of LAM. In the study, the subgroup analysis of diagnostic criteria revealed an insignificant difference in the sensitivity between definite and probable TBM (51% vs. 23%). In addition, meta-regression analysis supported the fact that LFA has a high sensitivity of 52% compared to the ELISA method (31%). However, more prospective studies are required to validate this finding.

The study has several limitations; thus, interpretation with caution is required. First, there are few primary studies on CSF LAM tests for TBM, and all the included studies were from sub-Saharan Africa, which may mean geographical bias. Therefore, the findings may not apply to other districts. Because sub-Saharan African countries suffer from HIV and TB epidemics, this special epidemic could enhance the applicability of the CSF LAM assay. Second, there was significant heterogeneity among the studies, which indicates that attention is required to interpret the results. Third, there are no clear consensus cutoff values for CSF LAM assays. Hence, urinary cutoff values were used for discrimination. A need to determine the optimal one may be required by ROC analysis. Fourth, due to limited studies, data on CD4 cell counts were not sufficient for subgroup analysis. Hence, further study is required to examine the association between CD4 cell count and diagnostic accuracy.

## Conclusion

Although more prospective studies are required to validate the CSF LAM assay’s diagnostic performance, current evidence supports that the diagnostic yield of the CSF LAM assay remains unsatisfactory for TBM detection. In addition, its performance may be enhanced by combining it with other methods (such as Xpert) or incorporating it into the Marais scoring system (4). Moreover, the protocol of the CSF LAM assay should be optimized with regard to CSF collection, preparation, and the optimal cutoff value.

## Data availability statement

The original contributions presented in the study are included in the article/Supplementary material, further inquiries can be directed to the corresponding authors.

## Author contributions

M-SW and Y-AZ designed the study and drafted the initial manuscript. Y-LC and M-MZ performed the screening and collected the data. C-PG supervised the data collection process. All authors contributed to the article and approved the submitted version.

## Funding

This project was supported by the Taishan Scholar Project of Shandong Province (NO.tsqn202211358).

## Conflict of interest

The authors declare that the research was conducted in the absence of any commercial or financial relationships that could be construed as a potential conflict of interest.

## Publisher’s note

All claims expressed in this article are solely those of the authors and do not necessarily represent those of their affiliated organizations, or those of the publisher, the editors and the reviewers. Any product that may be evaluated in this article, or claim that may be made by its manufacturer, is not guaranteed or endorsed by the publisher.
