# Supplementary material for: Diagnostic value of the cerebrospinal fluid lipoarabinomannan assay for tuberculous meningitis: a systematic review and meta-analysis
Source: Front Public Health. 2023 Sep 21;11:1228134. doi: 10.3389/fpubh.2023.1228134 (PMC10552265; doi:10.3389/fpubh.2023.1228134)
Supplement: Supplementary file 1 [file Data_Sheet_1.DOC]

**1. Electronic search strategy**

All search run: April 19^th^, 2023

**2. Databases (searching syntax; records, n)**

**2.1 PubMed (n=435)**

(Cerebrospinal OR CSF OR Meningeal OR ((Spinal OR Lumbar) AND (puncture OR tap)) OR ((tubercul* OR TB) AND mening*) OR (Tuberculosis, Meningeal) OR TBM) AND (lipoarabinomannan OR LAM OR Chemogen OR FujiLAM OR ((Abbott OR Alere) AND Clearview))

**2.2 Embase (n=696)**

(Cerebrospinal OR CSF OR Meningeal OR ((Spinal OR Lumbar) AND (puncture OR tap)) OR ((tubercul* OR TB) AND mening*) OR ('Tuberculosis, Meningeal') OR TBM) AND (lipoarabinomannan OR LAM OR Chemogen OR FujiLAM OR ((Abbott OR Alere) AND Clearview))

**2.3 Scopus (n=124)**

(Cerebrospinal OR CSF OR Meningeal OR ((Spinal OR Lumbar) AND (puncture OR tap)) OR ((tubercul* OR TB) AND mening*) OR ("Tuberculosis, Meningeal") OR TBM) AND (lipoarabinomannan OR LAM OR Chemogen OR FujiLAM OR ((Abbott OR Alere) AND Clearview))

**2.4 Web of Science (n=626)**

TS=(Cerebrospinal OR CSF OR Meningeal OR ((Spinal OR Lumbar) AND (puncture OR tap)) OR ((tubercul* OR TB) AND mening*) OR ("Tuberculosis, Meningeal") OR TBM) AND (lipoarabinomannan OR LAM OR Chemogen OR FujiLAM OR ((Abbott OR Alere) AND Clearview))

**2.5 CINAHL (n=74)**

(Cerebrospinal OR CSF OR Meningeal OR ((Spinal OR Lumbar) AND (puncture OR tap)) OR ((tubercul* OR TB) AND mening*) OR ("Tuberculosis, Meningeal") OR TBM) AND (lipoarabinomannan OR LAM OR Chemogen OR FujiLAM OR ((Abbott OR Alere) AND Clearview))

**2.6 Cochrane Library (n=27)**

(Cerebrospinal OR CSF OR Meningeal OR ((Spinal OR Lumbar) AND (puncture OR tap)) OR ((tubercul* OR TB) AND mening*) OR ("Tuberculosis, Meningeal") OR TBM) AND (lipoarabinomannan OR LAM OR Chemogen OR FujiLAM OR ((Abbott OR Alere) AND Clearview))
